# Next-generation fermented foods: engineering the future of functional nutrition and emerging food safety challenges

**DOI:** 10.3389/fmicb.2026.1869717

**Published:** 2026-06-16

**Authors:** Bersu Nur Hacıfettahoğlu, Saniye Bilici

**Affiliations:** 1 Department of Nutrition and Dietetics, Faculty of Health Sciences, Kastamonu University, Kastamonu, Türkiye; 2 Department of Nutrition and Dietetics, Faculty of Health Sciences, Gazi University, Ankara, Türkiye

**Keywords:** microbial risk assessment, novel fermented foods, precision fermentation, regulatory frameworks, starter cultures, synthetic biology

## Abstract

Fermented foods have long been recognized for their significant contributions to human health, particularly in enhancing gastrointestinal functionality, increasing microbial diversity, and improving nutrient bioavailability. In recent years, the intersection of traditional fermentation with advanced microbiology and synthetic biology has catalyzed the emergence of novel production techniques. The development of specialized starter cultures and the implementation of precision fermentation allow for unprecedented control over microbial interactions and the fermentation environment. These biotechnological innovations facilitate the production of foods with superior digestibility and enriched micronutrient profiles, yet they also introduce critical concerns regarding potential biochemical risks and consumer safety. The utilization of genomically modified microorganisms, including those developed through advanced Cas9-based approaches or intentional metabolic engineering, has sparked debate over regulatory oversight and long-term health implications. Beyond genetic concerns, the fermentation process can inadvertently lead to the formation of antinutrients and allergenic compounds, such as biogenic amines, mycotoxins, and advanced glycation end-products (AGEs). Furthermore, the potential presence of antibiotic resistance genes and pesticide residues in raw materials underscores the vulnerability of the production chain. This review critically evaluates the latest microbial selection strategies and biotechnological innovations, while emphasizing the imperative role of global regulatory bodies, such as the EFSA (European Food Safety Authority) and FDA (Food and Drug Administration), in establishing stringent safety standards. Ultimately, balancing technological advancement with comprehensive risk assessment is essential for the sustainable growth of the fermented food industry.

## Graphical Abstract

**Figure:**
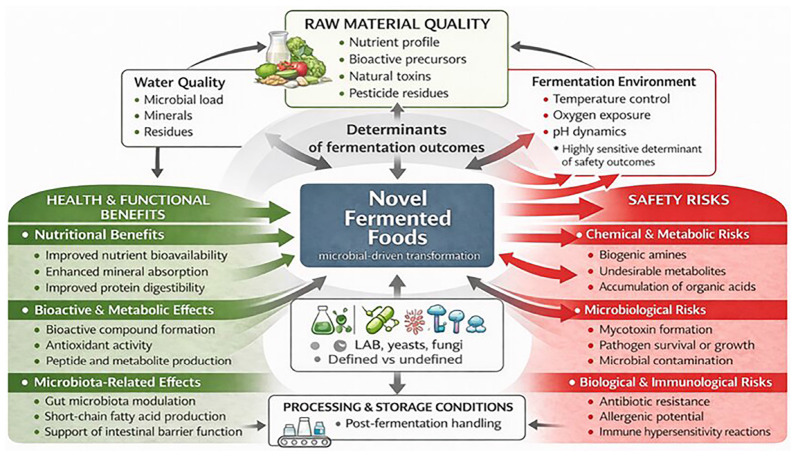
Overview of the major determinants shaping the quality, functional benefits, and safety risks of novel fermented foods.

## 1 Introduction

Fermentation is a foundational biotechnology historically utilized to enhance the shelf-life and organoleptic properties of food products. By improving microbial stability and safety, this process allows finished fermented foods to be stored effectively, often remaining stable even at ambient temperatures (Dimidi et al., 2019). The International Scientific Association for Probiotics and Prebiotics (ISAPP) defines fermented foods and beverages as foods produced through targeted microbial growth and enzymatic transformations of food components. This definition provides a clear distinction between fermented and non-fermented counterparts while emphasizing the transformative role of microorganisms in food biotechnology (Marco et al., 2021). Novel foods are broadly conceptualized within the Food and Agriculture Organization of the United Nations (FAO) and Codex Alimentarius system as “new food sources and production systems (NFPS),” encompassing foods derived from novel sources or produced using innovative technologies (Mukherjee and Fattori, 2022). A defining characteristic of these foods is their limited or absent history of human consumption, which necessitates tailored safety assessment approaches prior to market introduction (Mukherjee and Fattori, 2022). Therefore, this review aims to critically examine novel fermented foods in terms of their technological innovations, potential nutritional and functional benefits, and emerging safety challenges.

## 2 Literature search strategy

A narrative literature review was conducted to synthesize current evidence on novel fermented foods, with particular emphasis on microbial innovation, functional nutrition, food safety, and regulatory challenges. Relevant literature was searched in PubMed, Web of Science, and Google Scholar using broad and topic-specific search terms. The main search terms included “fermented foods,” “novel foods,” “novel fermented foods,” “novel fermentation,” “precision fermentation,” “starter cultures,” “microbial fermentation,” “synthetic biology,” “microbial engineering,” “functional foods,” and “food safety.” These terms were combined with Boolean operators such as “AND” and “OR” according to the focus of each subsection. Additional targeted searches were performed for specific safety and regulatory issues using terms such as “biogenic amines,” “mycotoxins,” “antibiotic resistance genes,” “allergenicity,” “GRAS,” “QPS,” “EFSA,” and “FDA.” Reference lists of relevant articles and reports were also manually screened to identify additional sources. Peer-reviewed original articles, review papers, and reports from scientific or regulatory organizations were considered. Publications were selected based on their relevance to the technological, nutritional, microbiological, safety-related, and regulatory aspects of novel fermented foods.

## 3 Definition and scope of novel foods

Within the European Union (EU) novel food framework, novel foods refer to foods and ingredients that had not been consumed to a significant degree within the EU before 15 May 1997, including products derived from fungi and algae, foods produced using nanotechnological processes, and ingredients without a previous history of human consumption (Turck et al., 2016; Ververis et al., 2020). Novel foods have become increasingly relevant in recent years in response to changing consumer preferences, sustainability concerns, the need to extend shelf life and reduce food spoilage, and the expansion of biotechnology-driven food production systems, including precision fermentation and microbiome-informed fermentation approaches (Mukherjee and Fattori, 2022; Teng et al., 2021; Augustin et al., 2024; Arrigan et al., 2024).

European Food Safety Authority (EFSA) published its first assessment of a novel food application in 2004 concerning the use of Enova oil, followed by its first evaluation of an insect-based novel food in 2021. EFSA has contributed to the scientific evaluation of novel foods through its regulatory and scientific activities, including recent assessments of emerging food technologies (EFSA, 2025). In addition, EFSA organized a Scientific Colloquium in 2023 on cell culture-derived foods and food ingredients, which addressed safety and methodological considerations relevant to risk assessment (Afonso et al., 2024). Novel foods are broadly conceptualized within the Food and Agriculture Organization of the United Nations (FAO) and Codex Alimentarius system as “new food sources and production systems” (NFPS), encompassing foods derived from novel sources or produced using innovative technologies (Table 1). As summarized in Table 1, these categories include foods with modified or newly designed molecular structures, products obtained from microbial, fungal, algal, plant, animal, or cell-based sources, and foods produced through emerging technologies such as novel processing or nanotechnology. This classification highlights that novelty may arise from different dimensions rather than from a single defining feature. For instance, structural innovation refers to foods with altered or newly designed molecular characteristics, whereas biological-origin categories include products derived from microorganisms, fungi, algae, plants, animals, or cell and tissue cultures. Technological innovation, on the other hand, emphasizes the role of emerging production methods that may modify the composition, structure, or nutritional properties of foods. Non-traditional materials and nanotechnology-based applications further expand the scope of novel foods by introducing ingredients or structures with limited previous dietary exposure. In this context, novel fermented foods may overlap with several of these categories, particularly when they involve unconventional substrates, microbial or fungal sources, engineered microorganisms, or fermentation processes that substantially alter the final product.

**TABLE 1:** Overview of food categories covered under the EU novel food framework.

Category	Type/source	Explanation
Structural innovation	Modified or newly designed structures	Foods characterized by a deliberately altered or entirely new molecular composition not previously consumed as food
Biological origin	Microbial, fungal or algal sources	Products obtained from or produced using microorganisms, fungi, or algae
	Plant-derived sources	Foods originating from plants without established safe consumption history or produced via non-conventional propagation techniques
	Animal-derived sources	Foods obtained from animals, excluding those with a documented history of safe use through conventional breeding
	Cell-based production	Foods developed from cell or tissue cultures derived from plants, animals, or microorganisms
Technological innovation	Emerging production methods	Foods produced through novel processing techniques that may alter their composition, structure, or nutritional properties
Non-traditional materials	Mineral origin	Products derived from inorganic (mineral) sources used as food ingredients
Advanced applications	Nanotechnology-based foods	Foods incorporating engineered nanomaterials
Regulatory extension	Expanded use beyond supplements	Ingredients previously restricted to food supplements but now intended for inclusion in other food categories

Compiled by the authors based on Turck et al. (2016).

Novel fermented foods support the definitions of fermented foods and novel foods. Novel fermented foods can be understood within the framework of “novel fermentation,” defined as the integration of traditional fermentation practices with rational microbiome design strategies, enabling the development of innovative fermented products (Arrigan et al., 2024). Consequently, they are often considered within the broader novel foods framework, as their limited history of consumption and innovative production processes may require case-by-case safety evaluation prior to market introduction (Dimidi et al., 2019; Marco et al., 2021). Therefore fermented foods are characterized by controlled microbial activity, whereas novel foods are defined by their limited or absent history of human consumption. Novel fermented foods on the other hand occupy an intersectional position, combining microbial transformation processes with innovative production approaches, such as novel substrates, engineered microorganisms, or advanced fermentation technologies.

The landscape of fermented food production has shifted from traditional, experience-based methods toward advanced biotechnological approaches. Conventional processes, such as spontaneous fermentation and backslopping, often depend on naturally occurring microbial communities and may therefore result in variability in microbial composition, product quality, and functional outcomes (Whittington et al., 2019). In contrast, recent developments in food biotechnology have enabled more controlled fermentation systems through defined starter cultures, precision fermentation, microbiome-based design, and the use of alternative substrates. These approaches allow greater control over microbial activity, substrate transformation, metabolite production, and product consistency. As summarized in Table 2, novel fermentation systems include several complementary strategies. Precision fermentation uses selected or optimized microorganisms to produce specific food ingredients under controlled conditions. Plant-based protein and novel substrate fermentation aim to improve digestibility, reduce antinutritional factors, and generate bioactive compounds from alternative raw materials. Synthetic microbial consortia and starter culture innovation focus on controlling microbial interactions and increasing reproducibility, whereas functional fermentation is designed to enhance specific health-related properties through microbial metabolites. Upcycling fermentation also contributes to sustainability by converting food by-products into value-added ingredients. Therefore, novel fermentation should be considered not as a single technique, but as a broad technological framework combining microbial control, substrate diversification, functional targeting, and sustainability-oriented production.

**TABLE 2:** Conceptual classification of novel fermented foods based on technological innovations and functional objectives.

Category of novel fermentation approach	Innovation dimension	Description	Representative applications	Primary purpose of fermentation	References
Precision fermentation	Microbial engineering	Application of genetically engineered or metabolically optimized microorganisms for controlled biosynthesis of food components	Microbial production of dairy proteins and food ingredients	Production of targeted compounds with controlled composition and improved sustainability	Hilgendorf et al., 2024; Teng et al., 2021; Augustin et al., 2024
Plant-based protein fermentation	Substrate diversification	Fermentation of plant-derived proteins using microbial systems to enhance nutritional and functional properties	Fermented pea, soy, and plant-based protein analogs	Enhancement of protein digestibility and generation of bioactive peptides	Fan et al., 2025; Farid et al., 2024; Dhiman et al., 2025
Synthetic microbial consortia	Microbiome design	Use of designed or optimized microbial communities to control fermentation processes	Multi-strain fermentation systems with defined microbial interactions	Improvement of fermentation consistency, safety, and metabolic functionality	Arrigan et al., 2024; Louw et al., 2023
Novel substrate fermentation	Substrate diversification	Use of non-traditional raw materials in fermentation processes	Fermented quinoa, sorghum, and cereal-based products	Reduction of antinutritional factors and improvement of nutrient bioavailability	Kitessa, 2024
Functional fermentation	Functional targeting	Fermentation processes designed to deliver specific health-related benefits through microbial metabolites	Fermented tea-based beverages, kefir, probiotic dairy products	Modulation of gut microbiota, oxidative balance, and immune-related responses	Bonifacio et al., 2025; Lawrence et al., 2025; Sybesma et al., 2025; West et al., 2025
Starter culture innovation	Process optimization	Development and application of tailored starter cultures with defined functional and safety properties	Selection and optimization of lactic acid bacteria strains	Enhancement of product stability, safety, and functional consistency	Gänzle et al., 2024; Comunian and Chessa, 2024
Upcycling fermentation	Sustainability-driven processing	Utilization of food by-products and waste streams as substrates in fermentation	Fermentation of agro-industrial by-products such as bran and spent grains	Valorization of waste streams and improvement of nutritional and environmental sustainability	Ajanaku et al., 2025

## 4 Microbial dynamics and ecological complexity in fermented foods

Fermentation is among the oldest techniques used for extended food preservation and involves the use of microorganisms. Fermented food matrices serve as hosts to intricate microbial ecosystems, characterized by diverse consortia of beneficial microorganisms, including lactic acid bacteria (LAB), acetic acid bacteria, yeasts, and filamentous fungi. These microbial communities are inherently non-static; they undergo continuous successional evolution throughout the fermentation period in response to a complex interplay of environmental variables. Specifically, these populations dynamically adapt to intrinsic parameters (substrate composition, pH changes, and nutrient kinetics) as well as extrinsic factors including thermal conditions, atmospheric oxygen tension, and the duration of the fermentation cycle (Park and Mannaa, 2025; Sharma et al., 2020).

The function of microorganisms in fermentation involves the decomposition of complex nutritional components (carbohydrates and other macromolecules) into simpler compounds. These transformations take place in the presence of various beneficial catabolites, including B vitamins, minerals, and omega-3 fatty acids (Siddiqui et al., 2023). However, fermentation is not solely utilized for prolonging the shelf life of food; it is also deeply embedded in cultural and religious traditions. This is one of the reasons why fermentation is not only used in production processes but is also transmitted across generations (Anyogu et al., 2021). In some nations, the implementation of fermentation techniques is essential due to challenging harvest periods or insufficient transportation infrastructure. This brings out the need for fermented foods. Other purposes of fermentation include the reduction of antinutrients or toxic components naturally present in raw materials, as well as the production of alcohol and sauces (Gänzle et al., 2024). Since sugars are metabolized, fermented foods may also contain fewer calories than the raw materials in some cases (Teng et al., 2021).

A broad range of raw materials is employed in fermentation processes. Cereals, dairy products, various herbs, legumes, meat, fish, as well as different vegetables and fruits, are utilized, resulting in the production of various types of fermented foods. Increased food diversity might encourage people to try out new things and increase fermented food consumption (Tamang et al., 2020).

The fermentation process is characterized by dynamic ecological successions governed by microbial competition and shifting environmental variables. Initial substrate colonization by diverse microorganisms is gradually replaced by the dominance of specialized, acid-tolerant groups as oxygen depletion and organic acid accumulation exert selective pressures. In vegetable fermentation, early aerobic activity facilitates the establishment of LAB, whose acid production suppresses pathogens and ensures microbial safety. These successional shifts are fundamental to the final product’s sensory profile and shelf life, highlighting the necessity of controlled ecological dynamics for maintaining consistent food quality (Filannino et al., 2018; Wolfe and Dutton, 2015).

Next-generation sequencing (NGS) and metagenomics have significantly refined our understanding of the microbial diversity within fermented foods by providing high-resolution profiling beyond traditional methods. These molecular advancements reveal that specific microbial consortia are precisely shaped by the biochemical nature of the substrate, environmental parameters, and the selection of starter cultures. Consequently, these tools offer a critical framework for ensuring the microbial safety and functional consistency of the fermentation process (Tigrero-Vaca et al., 2025).

### 4.1 Technological innovations: from traditional to novel fermented foods

Natural fermentation is driven by the dominance of fermentative microorganisms, which suppress pathogens through metabolite production and subsequent pH reduction. In traditional or small-scale production settings, this process is often facilitated by the addition of microbe-rich ingredients or the use of previously fermented products as an inoculum (Skowron et al., 2022). Recent technological advancements have transitioned the field toward standardized starter cultures and precision fermentation, integrating genetic and multi-omic approaches into the modern market.

With advancements in the microbiology discipline, starter cultures have been increasingly employed throughout fermentation processes, thereby facilitating the large-scale production of fermented foods. These starter cultures must be used under defined and controlled conditions within industrial manufacturing settings. The predominance of natural microbiota not only restricts the proliferation of undesirable microorganism species or strains but also diminishes the synthesis of toxic compounds by these microorganisms, thus improving food safety (Mannaa et al., 2021; Voidarou et al., 2020).

The predictability of spontaneous fermentation is primarily dictated by the nutritional and microbial composition of the raw material. To ensure process stability and inhibit spoilage, backslopping is utilized to establish dominant strains, often requiring precise environmental adjustments such as anaerobic conditions (Filannino et al., 2018; Mudoor Sooresh et al., 2023). By applying evolutionary biology and ecological frameworks, specifically dispersal, selection, drift, and diversification, it is now possible to steer microbial community assembly. This transition from traditional practice to ecological regulation facilitates a deeper understanding of microbial interactions, ultimately enabling the precise prediction of fermented product quality and nutritional value (Louw et al., 2023).

Starter cultures serve as critical regulatory agents in fermentation, standardizing microbial populations to ensure sensory consistency and process reliability. The transition from isolation, identifying high-quality reference strains, to large-scale biomass adaptation is a rigorous biotechnological trajectory. This systematic development is fundamental for scaling both traditional and novel fermented food production while mitigating fermentation defects. While starter cultures provide industrial scalability, they often fail to replicate complex fermentations, such as kefir and kombucha, that rely on surface-associated biofilms (Comunian and Chessa, 2024; Jimenez et al., 2022). This challenge is compounded by the emergence of novel fermented foods lacking traditional counterparts, necessitating specialized selection criteria for starter cultures to bridge the gap between historical microbial dynamics and modern food innovation (Muhammed et al., 2025). This highlights both the divergence and overlap between traditional and novel fermentation systems in terms of microbial control and functional outcomes. A comparative overview of these mechanistic differences is presented in Table 3.

**TABLE 3:** Mechanistic differences between traditional and novel fermentation systems.

Process step	Traditional fermentation	Novel/precision fermentation
Microbial initiation	Natural contamination or back-slopping	Deliberate inoculation with defined strains
Community assembly	Ecological succession driven by environment	Controlled assembly via strain selection/design
Selection pressure	pH, salt, temperature naturally select microbes	Artificially optimized conditions and strain engineering
Microbial interactions	Emergent, not fully predictable	Modeled and partially predictable
Metabolic output	Variable, depends on community dynamics	Targeted and optimized metabolite production
Process control	Indirect control via environment	Direct control via microbiome design and monitoring
Safety control	Relies on competitive exclusion and acidification	Relies on strain selection, screening, and regulation

Developed by the authors based on concepts reported in Arrigan et al. (2024), Comunian and Chessa (2024), Marco et al. (2021), and Wolfe and Dutton (2015).

Synthetic biology is revolutionizing starter culture development by enabling the precise genomic engineering of strains with bespoke functional traits. By identifying and manipulating specific metabolic pathways, these technologies streamline the selection process and facilitate the design of microbial strains with tailored performance characteristics (van Wyk et al., 2020). Clustered Regularly Interspaced Short Palindromic Repeats-Cas9 (CRISPR-Cas9) and related genetic tools have further contributed to starter culture development by enabling high-precision microbial genome editing. This technology has been increasingly explored for optimizing diverse strains, including yeast, thereby supporting the transition from laboratory manipulation to potential industrial application (Kumar et al., 2025). The primary objective is to facilitate high-precision genetic modifications in microorganisms, thereby optimizing their fermentative properties to enhance the overall quality and functional outcomes of fermented food products (Kanchan et al., 2026). The CRISPR/Cas9-mediated elimination of cryptic plasmids in the kimchi-associated *Leuconostoc citreum* strain represents a food-grade strategy for developing safe LAB devoid of residual antibiotic-resistance markers (Jang et al., 2017). Moreover, many CRISPR-based approaches in novel fermented food production remain largely confined to experimental or pilot-scale applications due to limited implementation, high development costs, and regulatory uncertainty. Although CRISPR-Cas9 has expanded the possibilities for optimizing microbial starter cultures, its safety profile in novel fermented food systems remains context-dependent and not fully established. To date, the available literature does not clearly identify a specific adverse human health pathway directly attributable to the consumption of CRISPR-Cas9-modified microorganisms. Instead, the safety concerns are mainly discussed as precautionary and mechanistic issues, including potential off-target editing, genetic instability, unintended metabolic alterations, possible changes in virulence- or resistance-associated traits, and the transferability of genetic elements under certain conditions (Eş et al., 2019; Rahman et al., 2025). Therefore, these microorganisms should not be considered inherently unsafe; however, they should also not be regarded as inherently risk-free. A case-by-case assessment is needed, focusing on the edited strain, the intended genetic modification, fermentation performance, metabolite profile, genetic stability, absence of transferable antibiotic resistance markers, and consistency under industrial production conditions (Ishii and Araki, 2016; Eş et al., 2019; Rahman et al., 2025).

By elucidating the genomic adaptations of microbial species to specific environments, recent research has established a robust framework for selecting *Lactobacillus* and *Leuconostoc* strains that optimize the stability and sensory profiles of novel fermented foods through the production of organic acids, carbon dioxide, and volatile aromatic compounds (Anumudu et al., 2024). Members of the *Bacillus subtilis* group are increasingly utilized in innovative fermented foods due to their ability to enhance nutrient bioavailability through protein and phytate hydrolysis, while simultaneously enriching sensory profiles via the enzymatic production of kokumi-associated γ-glutamyl dipeptides (Gao et al., 2025). Techniques such as metagenomics, metatranscriptomics, and metabolomics also provide valuable information on the genetic diversity and functional potential of microorganisms; thus, they assist in associating specific strains with antimicrobial compounds and resistance genes, as also discussed in the food safety section of this manuscript (Gänzle et al., 2024; Li et al., 2023). In a study involving genomic research and soybeans, a novel soy-based fermented food was developed through the use of *Bacillus amyloliquefaciens*, which was genetically modified to produce lycopene (Zou et al., 2022). Genomic research is highly complex, necessitating long-term observation and detailed analysis. It is challenging to focus on specific organisms and categorize them as beneficial or harmful due to their ongoing interactions, and it is even more difficult to detect genes because of variability in their expression (Ma et al., 2024).

An additional advancement in novel fermented food production is the use of fungal and yeast starter cultures. Traditionally employed in products such as bread, wine, and beer, fungal and yeast starters are increasingly applied in industrial systems to enhance aroma and functional properties, particularly in non-traditional substrates. These microorganisms can be sourced from diverse ecological types, including plants, fruits, and animal-derived environments representing a reservoir of untapped biodiversity (Chen et al., 2023; Hernández-Velázquez et al., 2024). Advances in biotechnology, such as hybridization, introgression, and interspecies gene transfer, have been shown to enable the development of optimized microbial strains, supporting improved fermentation performance and product quality (Gabaldon, 2020; Winans, 2022).

Process control of fermentation has developed with the advancements in microbiology and biology. But the relative cost profile also increased with the introduction of precision fermentation. Even though it is said that costs will decrease with more research in the industry, this must be noted as a limitation of novel fermented foods. But the better the microbial standardization, the lower the health risks. Therefore, it is possible to mention the use of starter cultures brought the fermented food market to a whole different point (Hilgendorf et al., 2024).

Advances in microbiology, molecular biology, and process engineering have introduced a higher level of control into fermented food production. Unlike traditional fermentation systems, which largely depend on naturally occurring microbial communities and empirical practices, novel fermentation approaches allow the deliberate regulation of microbial composition, substrate transformation, environmental parameters, and metabolite production. This transition improves process reproducibility and may reduce safety risks by limiting undesirable microorganisms and batch-to-batch variability. However, these advantages are accompanied by higher production costs, greater technical complexity, and the need for advanced monitoring systems, particularly in precision fermentation. Therefore, the distinction between traditional and novel fermentation systems should be evaluated not only in terms of technological advancement, but also in relation to process control, scalability, cost, safety, and functional predictability. The main mechanistic differences between these two fermentation systems are summarized in Table 3.

### 4.2 Microbial selection and biotechnology

A diverse spectrum of microorganisms, ranging from bacteria like lactic acid and *Bacillus* species to yeasts and filamentous fungi, plays a critical role in food fermentation, where they function as either primary or secondary fermenters across various dairy, meat, and soy-based products (Tamang et al., 2020). The selection of microorganisms and starter cultures depends on their technological functionality, traditional use, and safety profile. Regulatory bodies such as EFSA and FDA (Food and Drug Administration) assess safety through Qualified Presumption of Safety (QPS) and Generally Recognized as Safe (GRAS) frameworks, respectively, and regularly update approved microorganism lists (EFSA et al., 2025). Safety evaluations are based on whole-genome sequencing for taxonomic identification, documented safe use, and the absence of virulence factors, biogenic amine production, and transferable antimicrobial resistance (Tsanasidou et al., 2021). It is crucial that microorganisms and starter cultures used in fermented food production have been tested and approved by regulatory authorities, particularly concerning specific health risks.

Traditional knowledge systems assist in choosing starter cultures for novel fermented foods. In traditional fermented foods, certain microorganisms are dominant in specific substrates. These microorganisms can also be used in the production of novel fermented foods. The information on microorganisms’ preferred environment can also be used in production (Hernández-Velázquez et al., 2024; Kahraman Ilıkkan et al., 2025). For instance, *Tetragenococcus halophilus* thrives in high-salt environments, *Oenococcus oeni* is well adapted to fermentations characterized by low pH and elevated ethanol levels, and *Staphylococcus* species tend to predominate in protein-rich matrices (Gänzle et al., 2024).

Another criterion is scientific knowledge from research. LAB, which are Gram-positive and non-spore-forming, play a central role in fermented foods by contributing to preservation through organic acid production and enhancing flavor via amino acid metabolism. LAB are prevalent in most fermented foods, including dairy, meat, cereal, and vegetable products (Ali et al., 2025). Certain heterofermentative species additionally produce carbon dioxide and acetate, while LAB can synthesize bacteriocins capable of inhibiting pathogens such as *Listeria monocytogenes* (Zapaśnik et al., 2022). Yeasts contribute to the production of CO_2_, ethanol, and volatile aroma compounds, whereas *Bacillus* spp. and filamentous fungi produce enzymes and antimicrobial metabolites (Malekijahan et al., 2025). Several microorganisms are increasingly being explored for emerging applications, including the fermentation of alternative protein substrates and plant-derived compounds (Fan et al., 2025).

Recent meta-omics studies show that fermented foods harbor dynamic, multispecies communities structured around a functional core microbiota. For example, cabbage fermentation follows a predictable succession from *Leuconostoc mesenteroides* to species such as *Lactiplantibacillus plantarum* and *Levilactobacillus brevis* (Wolfe and Dutton, 2015). The reactions initiated by microorganisms transform substrates into various metabolites contributing to flavor, aroma, texture, stability, and nutritional compounds associated with fermented foods. Management of cultures and ecological determinants plays a crucial role in the success of fermentation. Product quality parameters such as pH, temperature, and redox potential are influenced by raw material quality, salt concentration, microbial load, and environmental conditions (Sawant et al., 2025). In novel fermented food production, microbial selection can be guided by both traditional knowledge and contemporary biotechnological research. Innovative approaches enable the adaptation of traditional fermented products (e.g., fish- or cereal-based sauces such as garum, gochujang, and kharoli) across different regions, while fermentation of alternative substrates, including gluten-free cereals, provides valuable microbial insights for the development of next-generation fermented foods (Arrigan et al., 2024).

## 5 Functional and nutritional properties of novel fermented foods

The growing demand for plant-based and allergen-free foods has accelerated the development of novel fermented products aimed at improving nutritional quality while maintaining sensory properties (Farid et al., 2024). Sustainability and increasing demand have increased the importance of plant proteins. Fermentation improves protein bioavailability and enables the production of bioactive peptides. Crops such as barley and sorghum provide beneficial polyphenols and β-glucans but may require fermentation to overcome specific amino acid limitations. Consequently, plant fermentation has expanded beyond meat and dairy alternatives to include cereals, fruits, vegetables, and cocoa products (Zhao L. et al., 2024).

Recent advances in fermentation enhance sustainability by upcycling nutrient-rich by-products, such as brewer’s grains and fruit pulps. Precision fermentation and specialized starter cultures are being used to produce novel products with nutritional and organoleptic profiles. Biomass fermentation–derived cultured meat, for instance, is often preferred over plant-based alternatives due to its closer resemblance to animal meat (Augustin et al., 2024; Teng et al., 2021; Zhao L. et al., 2024). Precision fermentation may also contribute to environmental sustainability by reducing reliance on resource-intensive animal-derived ingredients. Life cycle assessment-based evidence indicates potential reductions in land use, water demand, and greenhouse gas emissions, particularly for animal-free dairy proteins; however, these benefits depend on substrate source, energy input, downstream processing, and production scale (Adeyeye et al., 2026; Manzoki et al., 2026). Therefore, the sustainability of precision fermentation-based novel foods should be evaluated case by case through life cycle assessment rather than assumed as an inherent advantage.

Algae, particularly freshwater species with high carbohydrate and amino acid contents and antioxidant activity, offer significant potential as novel substrates for fermented food development (Santiago-Díaz et al., 2022). In this context, algae-based systems fit within the broader framework of novel fermented foods because microbial fermentation may improve sensory properties, increase bioactive compound availability, and support sustainable food innovation. While promising for meat analogs, the application of algae is hindered by challenges in achieving meat-like textures and overcoming undesirable organoleptic properties. Consequently, refined cultivation methods and additional processing steps are essential to ensure sensory quality and consumer acceptance (Zhang et al., 2025).

Several ingredients produced using innovative fermented food techniques are currently available on the market. These ingredients include microbially derived steviol glycosides used as sweeteners, the synthesis of soy leghemoglobin in engineered Pichia pastoris utilized in plant-based burgers, animal-free egg replacers, and dairy proteins. These products demonstrate how the novel fermented food market has expanded in response to consumer demands such as vegetarian, vegan, or diabetic-friendly options (Augustin et al., 2024).

The fermentation of polyphenols with specific probiotic strains enhances antioxidant capacity and shelf life while increasing bioavailability through the breakdown of complex molecules. This process also enables selective microbial control by inhibiting pathogens, though the effectiveness of these interactions depends strictly on the specific polyphenol-probiotic pairing used in functional food development (Sharma et al., 2022).

## 6 The health-promoting landscape of emerging novel fermented foods

**FIGURE 1:**
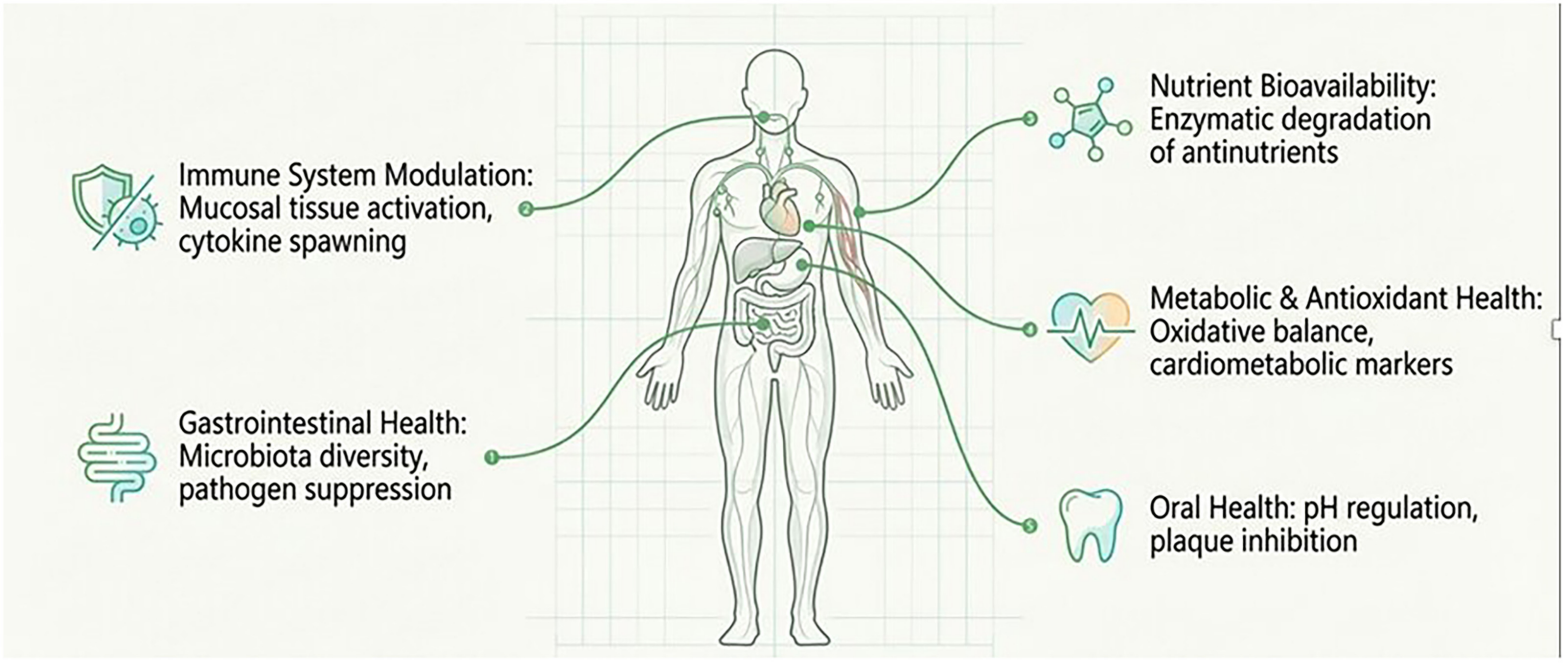
Health-promoting potential of fermented foods across multiple physiological domains.

Emerging evidence from controlled trials further supports the health-promoting potential of fermented foods across multiple physiological domains (Figure 1). Both fermented and novel fermented foods have been shown to support immune function, gastrointestinal health, and metabolic regulation (Bonifacio et al., 2025; Sybesma et al., 2025; West et al., 2025). Figure 1 summarizes the potential health-related effects of fermented foods across gastrointestinal, immune, metabolic, nutritional, and oral health domains. These effects are generally attributed to microbial activity, fermentation-derived metabolites, and biochemical changes occurring during fermentation, such as the degradation of antinutritional factors and the formation of bioactive compounds. Nevertheless, the health impact of fermented foods should not be generalized, as it may vary according to the food matrix, microbial composition, fermentation process, and strength of clinical evidence.

### 6.1 Nutritional benefits

During fermentation, microbial activities may significantly alter the nutritional composition, bioavailability, and physiological effects of fermented foods. Production of various types of bioactive metabolites increases the nutritional quality of the fermented product. The bioavailability of flavonoids and tannins is also enhanced through the transformation of phenolic compounds. With the breaking down of complex molecules to simpler forms, the bioavailability of components increases (Kitessa, 2024). Furthermore, fermentation can enhance the production of vitamins, organic acids, and various bioactive metabolites. Additionally, fermentation can decrease the amount of antinutritional factors (Sawant et al., 2025). For example, phytates and oxalates, antinutrients reducing bioavailability of minerals such as iron, zinc, and calcium, can be degraded through fermentation. In plant-based foods, mineral absorption is low due to their binding to non-digestible compounds, which can be decreased through fermentation (Dhiman et al., 2025).

### 6.2 Bioactive and metabolic effects

Fermented foods are highly regarded for their rich composition of viable microorganisms, microbial metabolites, and bioactive compounds. Fermented foods confer significant health benefits and facilitate vitamin synthesis and immune system regulation, thereby reducing the risk of gastrointestinal disorders, allergies, and inflammatory conditions (Park and Mannaa, 2025; Pyo et al., 2024).

Fermented foods may contain probiotics. Probiotics generate chemokines, cytokines, growth factors, and immunoglobulins, stimulating the immune system in the process. Notably, probiotic bacteria predominantly activate the mucosa-associated lymphoid tissue, supporting immune functions. However, evidence from fermented foods lacking defined probiotic strains suggests potential benefits for gut function and immune regulation, though data remain limited and are influenced by the food matrix (Dimidi et al., 2019). Advances in genomic and metabolite-based screening now allow the identification of strains with immunomodulatory properties, and many novel fermented products combine live microbes with minimally processed, fiber-rich plant materials that align with dietary patterns linked to lower chronic disease risk (Gänzle et al., 2024).

In addition, fermentation can influence the concentration of mycotoxins present in food products. LAB produce antifungal metabolites that reduce mycotoxin synthesis, while competitive nutrient utilization and environmental modifications further inhibit fungal growth. Bacterial cell-wall components—such as polysaccharides and peptidoglycans—can also interact with and sequester mycotoxins. Moreover, the use of starter cultures enables the selection of specific strains, thereby reducing mycotoxin production in fermented foods such as koji and cheese (Fayyaz et al., 2022; Skowron et al., 2022; Zapaśnik et al., 2022).

Finally, a specific health benefit of novel fermented foods is the manipulation of microorganisms and the regulation of the fermentation environment. The ability to target specific microorganisms reduces the presence of potential spoilage microorganisms. Additionally, novel fermentation enables the utilization of certain spoilage microorganisms in the fermentation process. Improved organoleptic qualities and enhanced nutritional content of fermented foods may increase consumer acceptance of novel fermented products concerning health benefits (Ajanaku et al., 2025).

### 6.3 Microbiota-related effects

Fermented foods represent a diverse category capable of delivering probiotics, prebiotics, synbiotics, and postbiotics, whether through live microbial consortia or non-viable components. However, their functional classification as “biotics” depends strictly on the level of microbial characterization, the reproducibility of the production process, and the rigor of clinical evidence supporting their specific health claims (Vinderola et al., 2023).

In addition, fermented foods contain probiotics, LAB, yeast, organic acids, ethanol, or antimicrobial compounds, which help balance the gut microbiome and improve digestive health (Latif et al., 2024). The physiological impact of fermented food consumption is primarily driven by the dynamic interactions between food-derived microbes and the host’s endogenous microbiota (Terpou et al., 2025). Moreover, emerging work also suggests that fermented food consumption may enhance microbial diversity and reduce inflammatory markers. In this context, studies demonstrate that microbiota, especially LAB, can colonize the gastrointestinal tract temporarily due to their tolerance to acid and bile. These bacteria are capable of synthesizing biologically active compounds, suppressing pathogens within the gastrointestinal tract, and modulating immunity through the epithelium (Dikbas et al., 2025; Wastyk et al., 2021; Zhao J. et al., 2024).

Furthermore, increased intake of fermented foods has been associated with modifications in oral microbiota. Fermented food consumption may reduce the pH of the oral mucosa and have a positive effect against oral inflammation. Additionally, other positive impacts on oral microbiota include the reduction of dental caries and periodontal disease, as well as the promotion of antioxidant production, which helps inhibit plaque growth (Skowron et al., 2022). Beyond these microbiota-related effects, randomized controlled human studies have investigated fermented foods in relation to immune function, oxidative stress, cardiometabolic markers, sleep-related outcomes, gut microbiota composition, and cognitive performance. Representative studies are summarized in Table 4.

**TABLE 4:** Examples of randomized controlled human studies on fermented foods and their functional effects.

Fermented food	Microorganism present	Beneficial effect associated	Study duration	Study group	References
Fermented milk (Qingrun)	*Bifidobacterium animalis* subsp. *lactis* Bl-04, *Lacticaseibacillus casei* formerly *Lactobacillus casei*, *Lactobacillus delbrueckii* subsp. *bulgaricus*, *Streptococcus thermophilus*.	Reduced incidence, duration, and severity of upper respiratory tract infections; improved immune function	12 weeks	Adults, *N* = 123 (Qingrun = 62, control yogurt = 61)	(Zhang et al., 2021)
Soy Kori-tofu	Not applicable / no live microbial strain reported; Kori-tofu protein was used as a fermented soy-derived ingredient.	Improvement in cardiometabolic markers within the intervention, but no significant difference compared to control	4 weeks	Adults aged 40–70 years, *N* = 45 (Kori-tofu = 22, control = 23)	(van den Belt et al., 2023)
Kefir	Mixed kefir microbiota, including lactic acid bacteria and yeasts; exact strain names were not reported.	Reduced awake time during sleep; modulation of gut microbiota	6 weeks	Children aged 8–13 years with ADHD, *N* = 53 (kefir = 22, placebo = 31)	(Lawrence et al., 2025)
Kombucha	Green tea kombucha microbial consortium; exact microbial species or strain names were not specified.	Reduced hydrogen peroxide levels	10 weeks	Adults with excess body weight, *N* = 59 (kombucha = 30, control = 29)	(Bonifacio et al., 2025)
Probiotic yogurt	*Lacticaseibacillus rhamnosus* yoba 2012 and *Streptococcus thermophilus* C106.	Reduced RTI symptoms over time; increased fecal bacterial load (no significant between-group differences)	9 weeks	Children aged 3–6 years, *N* = 196 (yogurt = 101, placebo = 95)	(Sybesma et al., 2025)
Soy	Non-probiotic fermented soy powder; exact microbial strains were not disclosed and no live cultures were reported.	Improved memory performance; improvement in global cognition in older women	12 weeks	Older adults ( ≥ 65 years), *N* = 47 (soy = 25, placebo = 22)	(West et al., 2025)

Overall, the studies summarized in Table 4 indicate that fermented food interventions may influence immune, oxidative stress-related, cardiometabolic, sleep-related, microbiota-related, and cognitive outcomes. However, these effects should be interpreted cautiously because the interventions differ in food matrix, microbial composition, intervention duration, and target population. In particular, strain-level information was not consistently reported across studies. Therefore, where available, exact microbial strain names were included in the table, while products without disclosed strain-level information were described more generally.

**FIGURE 2:**
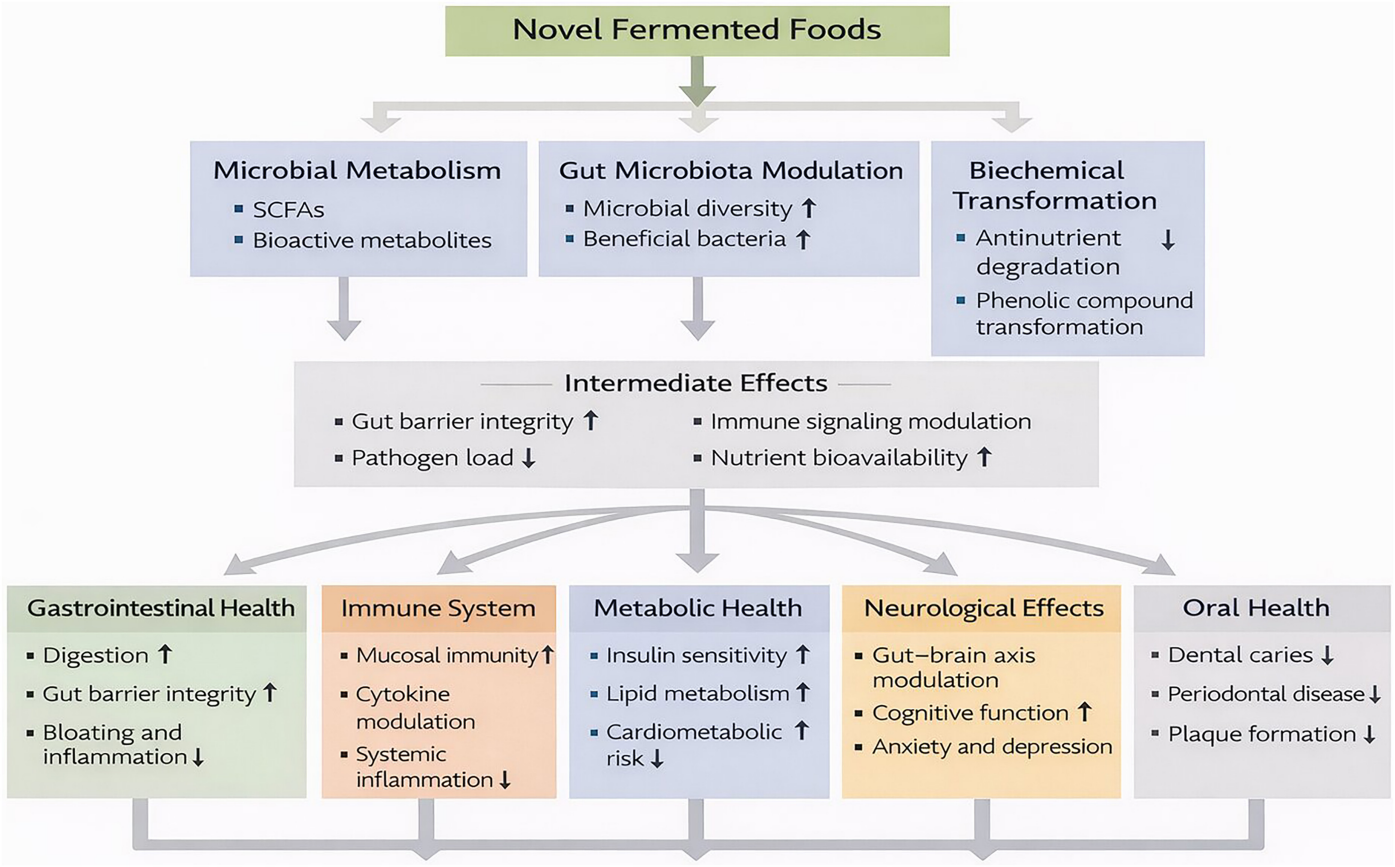
Mechanisms and health effects of novel fermented foods.

As discussed above, the health benefits of novel fermented foods are primarily mediated through key mechanisms, including modulation of the gut microbiota and biochemical transformation of food components during fermentation. Through these processes, fermentation may enhance nutrient bioavailability and generate bioactive metabolites that contribute to host health (Figure 2). Figure 2 summarizes how these mechanisms may act together rather than independently. Microbial metabolism can lead to the production of short-chain fatty acids and other bioactive metabolites, while changes in the gut microbiota may support microbial diversity and beneficial bacterial populations. At the same time, biochemical transformation during fermentation can reduce antinutritional factors and modify phenolic compounds. These changes may contribute to improved gut barrier function, immune signaling, nutrient availability, and pathogen control, which are then reflected in broader gastrointestinal, metabolic, immune, neurological, and oral health outcomes.

## 7 Risks and safety concerns

The safety of novel fermented foods should be evaluated through a comprehensive framework that considers chemical, metabolic, microbiological, biological, and immunological risks. These risks are influenced by raw material quality, microbial composition, fermentation conditions, processing technologies, storage practices, and consumer susceptibility. Fermented foods are often characterized by organic acid production, reduced pH, and the presence of antimicrobial metabolites; in some products, additional factors such as reduced water activity may further contribute to microbial stability. These properties have historically supported their perception as microbiologically safe. Similarly, alcoholic beverages (≥4% alcohol, pH < 4.5) are generally considered stable. However, safety is not universal, as fermentation may occur under spontaneous or poorly controlled conditions without defined starter cultures. Thus, while novel fermentation and novel fermented foods offer important benefits, they may also pose potential health risks (Teng et al., 2021). It is also important to note that individuals may have concerns regarding novel fermented foods, including their long-term usage, potential side effects, allergenic ingredients, and varying levels of acceptance or security concerns related to new technological developments. Since novel fermented foods are a rather new area, it is too early to talk about long-term usage and consumer behavior (Hilgendorf et al., 2024).

### 7.1 Chemical and metabolic risks

One health concern associated with fermented foods is their salt and alcohol content, which should be consumed in moderation. Fruit- and vegetable-based fermented foods may contain pesticide residues, while raw materials can be contaminated with heavy metals, underscoring the importance of sourcing from reliable suppliers (Wang et al., 2024). Certain nutrient or antinutrient compounds that arise during fermentation processes may pose a risk to food safety.

Histamine, tyramine, and other biogenic amines can be formed during the production of various fermented foods, including cheese, meat products, vegetables, soy-based foods, and wine. When ingested by individuals with particular allergies or compromised detoxification systems, it may lead to adverse effects. Biogenic amines (BAs) are produced during fermentation mainly via amino acid decarboxylation, with their formation influenced by raw material composition, microbial activity, fermentation duration, and environmental conditions (Marco et al., 2021). Elevated BA levels are associated with poor quality and spoilage, and may result from inadequate process control, including unfavorable environmental conditions and raw material characteristics (Zinina et al., 2022).

Cereal and legume-based novel fermented foods may contain other antinutrients such as lectins, tannins, and oxalates. Digestion of these antinutrients may result in side effects such as nausea and diarrhea (Wang et al., 2024). Another compound that presents a food safety concern is the formation of advanced glycation end-products (AGEs). AGE production is mostly because of the fermentation environment. With the production of nutrients like reducing sugars and free amino groups, it is possible for non-enzymatic glycation reactions. Although fermentation typically occurs at low temperatures, prolonged processing and subsequent thermal treatments may increase AGE formation, which has been associated with cancer, cardiovascular diseases, and chronic kidney disease, highlighting the need for controlled fermentation processes (Wang et al., 2025).

The discussion of mycotoxins was presented in the previous section. Research has shown that mycotoxins can exert adverse effects, especially in the gastrointestinal tract. Additional microbial metabolites produced during fermentation include citrulline and reuterin. These metabolites are precursors to toxic compounds such as ethyl carbamate and acrolein, respectively. However, the impact of these metabolites on health concerning fermented food consumption remains uncertain (Fitsum et al., 2025).

### 7.2 Microbiological risks

The use of inferior-quality products, combined with inadequate hygienic conditions, poses significant food safety risks, particularly in low- and middle-income countries and small-scale or household production settings. In these regions, higher-quality products are often exported or reserved for economic purposes, leaving lower-quality products for domestic consumption. This situation may promote the proliferation of pathogenic microorganisms or toxin formation, leading to foodborne illnesses and potential outbreaks (Fayyaz et al., 2022).

In line with these concerns, deficient production procedures and non-optimal storage conditions further negatively influence the quality of the product. This lowers the quality standards and may cause food safety issues. Various regions worldwide, especially in Africa and Asia, have reported the presence of different pathogens, including enterotoxicogenic and enterohemorrhagic strains. It is important to follow quality standards while buying raw materials and fermented foods themselves. Cross-contamination of the product after production also remains a possibility. Among the reported pathogens are *Escherichia coli*, *Shigella* spp., *Salmonella* spp., Enterotoxigenic *Staphylococcus aureus*, *Listeria monocytogenes*, and *Bacillus cereus* (Tamang et al., 2020; Wang et al., 2024).

Fermented foods are generally regarded as safe, and reported cases of gastroenteritis remain rare, especially when production involves high-quality raw materials and appropriate hygienic conditions. However, cheese products and fermented foods with low acidity levels present potential pathogen hazards, including *Listeria monocytogenes*, *Salmonella*, and *Clostridium botulinum* (D’Ambrosio et al., 2026; Todorovic et al., 2024). The information on microorganisms related to traditional fermented foods also applies to novel fermented foods.

Beyond production and raw material quality, an additional concern in fermented food production is the quality of the water. It is important to check the water source in fermented food production, and the water must be devoid of microbial contamination. Water purification may also be used if available in the facilities. Limited water access, drought conditions, and contaminated water sources, particularly in low-income nations, exacerbate the risks of *E. coli* and *Salmonella strains* (Anyogu et al., 2021).

Similarly, in developing countries, there may be deficiencies concerning prerequisite programs such as Good Manufacturing Practices. This can result in production processes that are not sufficiently supervised. Consequently, the risk of using unsafe packaging materials, non-sterile equipment, inadequate personnel training, and producing products contaminated with impurities increases (Mukherjee and Fattori, 2022).

From a regulatory perspective, authorities investigate the application of microorganisms in the production of novel fermented foods. However, it should be noted that even if a specific strain is declared safe, some subgroups may still experience adverse effects after consumption. An example is *Lacticaseibacillus rhamnosus*, which is classified as “GRAS” by the FDA. Nevertheless, there have been reports of infections in immunocompromised individuals following its consumption. Additionally, some microorganisms used in traditional fermented food production have limited use in novel fermented foods due to their potential pathogenicity (EFSA et al., 2023; Gänzle et al., 2024).

In this context, ensuring the safety of novel fermentation requires rigorous regulatory oversight to assess microbial interactions and long-term health implications. While frameworks vary globally, they primarily focus on documenting strain safety to support classifications such as generally recognized as safe. The FDA permits improved strains provided they lack foreign DNA and antibiotic resistance genes and demonstrate genetic stability (Mannaa et al., 2021; Vilela, 2021). However, current approaches remain largely strain-focused, limiting their ability to address complex microbial communities.

To mitigate microbiological risks associated with fermented foods, microorganisms used in production must be classified as safe and subjected to rigorous regulatory evaluation. In this context, systems such as the FDA GRAS and the European Food Safety Authority QPS frameworks assess microbial strains based on their history of safe use, genetic stability, and absence of traits such as pathogenicity or transferable antibiotic resistance. These approaches aim to ensure that starter cultures and food-associated microorganisms do not pose safety concerns. However, current regulatory frameworks remain largely strain-focused, which may limit their applicability in addressing the complexity of microbial interactions in novel fermented food systems (EFSA et al., 2023; Heo et al., 2022).

### 7.3 Biological and immunological risks

Certain novel fermented food products, particularly those based on wheat, peanuts, or eggs, may contain allergenic substances capable of triggering adverse immune reactions in susceptible individuals. Therefore, clear and informative product labeling is essential to prevent unintended exposure and protect consumer safety.

Fermented foods pose a potential risk of harboring antibiotic or chemotherapeutic-resistant genes. This risk comes from the extensive usage of antibiotics in agriculture and livestock husbandry. It is essential that the quality of such products be thoroughly assessed before processing. Furthermore, the use of antibiotics must be clearly documented in technical specifications, and specific antibiotic and chemotherapeutic resistance genes should be examined whenever feasible (Skowron et al., 2022).

Beyond direct chemical and microbiological hazards, fermented foods may also exert complex biological and immunological effects, which require comprehensive evaluation. In this context, broader risk–benefit assessment approaches have been proposed. Supported by the European Green Deal, the PIMENTO initiative (COST Action CA20128) provides an open-access platform to standardize microbial communities and promote fermentation-driven innovations. This framework evaluates the impact of fermented foods on multiple health domains, including gut health, allergenicity, immunity, metabolic health, cardiovascular function, bone health, and cognitive outcomes (Todorovic et al., 2024).

**FIGURE 3:**
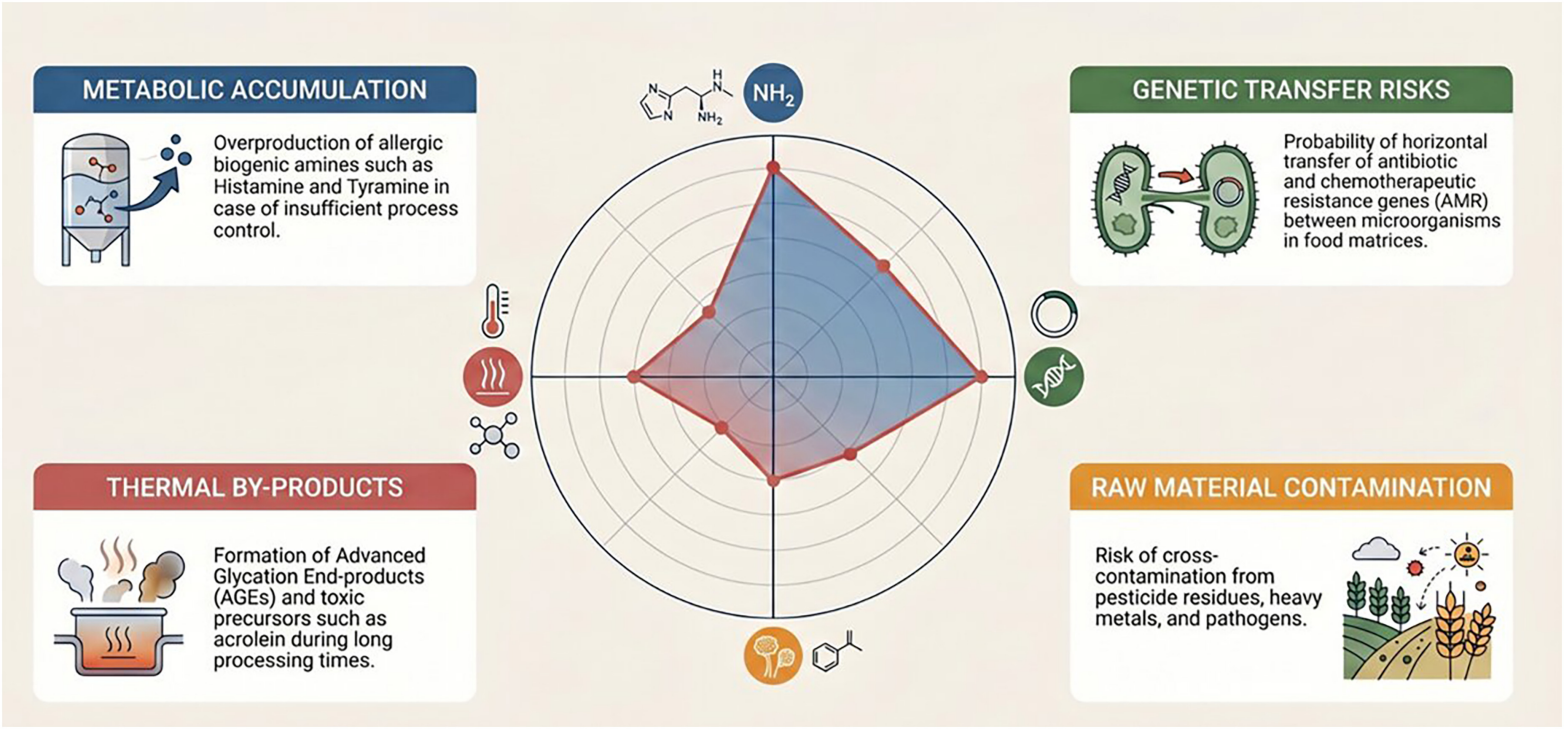
Mapping biochemical and microbiological risks of novel fermented foods.

Despite their potential benefits, novel fermented food products may also present safety risks that require careful evaluation. As illustrated in Figure 3, these risks may arise from different stages of the production chain, including raw material selection, microbial activity, fermentation control, and post-fermentation processing. Inadequate process control may contribute to the accumulation of biogenic amines, such as histamine and tyramine, while prolonged processing or subsequent heat treatment may promote the formation of AGEs and other toxic precursors. In addition, raw materials may introduce pesticides, heavy metals, pathogens, or other contaminants into the fermentation system. Another important concern is the possible presence or horizontal transfer of antibiotic and chemotherapeutic resistance genes among microorganisms used in or associated with fermentation. Therefore, the safety assessment of novel fermented foods should consider not only the selected microorganisms, but also raw material quality, processing conditions, microbial interactions, post-production handling, and regulatory monitoring.

## 8 Conclusion

The emergence of novel fermented foods represents a significant advancement in sustainable nutrition, offering enhanced digestibility through the conversion of macronutrients and promoting a more diverse gut microbiota. While these products support the growth of beneficial microorganisms, it is crucial to recognize that technological control and predictable outcomes do not inherently guarantee superior health profiles. Comprehensive risk assessments are essential to understand the complex microbial and metabolic interactions that occur within these novel matrices, ensuring that the shift toward innovation does not overlook potential safety concerns. Despite their potential, these foods carry specific risks, including the production of antinutrients like BAs and mycotoxins, as well as the presence of antibiotic-resistant genes or pesticide residues. The integration of advanced biotechnologies, such as CRISPR-modified strains, further necessitates transparent regulatory frameworks that evaluate the final product as a whole rather than focusing solely on individual microbial characteristics. Ultimately, achieving a safe and sustainable food system requires a balance between technological innovation and rigorous safety standards, supported by long-term human intervention trials to fully elucidate the metabolic impact of novel fermentation processes.
